# Rehabilitation for complicated dysphagia after synchronous head-and-neck and esophageal cancer surgery: A case report

**DOI:** 10.1097/MD.0000000000040338

**Published:** 2024-11-08

**Authors:** Xinyuan Xue, Amerull Azman, Cuicui Zhang, Yangjia Chen, Jun Ni, Zhi-Yong Wang

**Affiliations:** a Department of Rehabilitation, The First Affiliated Hospital, Fujian Medical University, Fuzhou, China; b Department of Rehabilitation, National Regional Medical Center, Binhai Campus of the First Affiliated Hospital, Fujian Medical University, Fuzhou, China.

**Keywords:** case report, deglutition disorders, esophageal neoplasms, head and neck neoplasms, rehabilitation

## Abstract

**Rationale:**

Surgical intervention for synchronous head-and-neck and esophageal cancers often results in complex dysphagia, significantly affecting postoperative quality of life. Swallowing dysfunction may become permanent or worsen, with potential impacts on noncancer-related mortality.

**Patient concerns:**

We report a rare case of multiple synchronous squamous cell carcinomas of the head and neck (tonsillar and epiglottic cancer) along with esophageal cancer, presenting for dysphagia rehabilitation following surgery.

**Diagnoses:**

Comprehensive evaluations—including magnetic resonance imaging, laryngoscopy, gastroscopy, and histopathology—led to diagnoses of left tonsil cancer (squamous cell carcinoma, T2N2bM0), epiglottic cancer (squamous cell carcinoma, T1N2bM0), and lower esophageal cancer (squamous cell carcinoma, T2N0M0). Postoperative videofluoroscopic swallowing study identified an anastomotic stricture at the level of the fifth cervical vertebra.

**Interventions:**

The patient underwent an 8-week rehabilitation program incorporating stretching exercises, swallowing behavior therapy, super-supraglottic swallow techniques, catheter balloon dilation, electrical stimulation, and respiratory therapy.

**Outcomes:**

Following rehabilitation, the patient was able to resume partial oral intake without aspiration, with significant improvement in anastomotic stricture and swallowing function.

**Lessons:**

This case of dysphagia underscores the anastomotic stenosis resulting from oncological surgical intervention. Dysphagia is a frequent complication in patients with synchronous head-and-neck and esophageal cancers. Comprehensive rehabilitation and assessment of swallowing function enabled safe oral intake postoperatively in this patient.

## 1. Introduction

Head and neck cancer is the sixth most common malignant tumor worldwide, accounting for approximately 4.8% of all systemic malignancies. Head and neck cancer can be complicated by secondary primary tumors in the entire aerodigestive tract, with an annual incidence of 3%.^[1]^ Treatments for synchronous head-and-neck and esophageal cancer include surgery, radiotherapy, and chemotherapy. Common complications after treatment include dysphagia, pharyngeal or esophageal stenosis, dry mouth, difficulty in opening the mouth, sensory disturbance, hoarseness, mucositis, caries, osteoradionecrosis, and pain. Swallowing dysfunction might be permanent or deteriorate,^[2]^ leading to malnutrition, aspiration pneumonia, and other diseases, and it could have a significant effect on non-cancer-related mortality. In this paper, we report a rare case of multiple head and neck squamous cell carcinomas (tonsillar cancer, epiglottis cancer) with synchronous esophageal cancer after surgery for dysphagia rehabilitation.

## 2. Medical history

**Figure 1.**
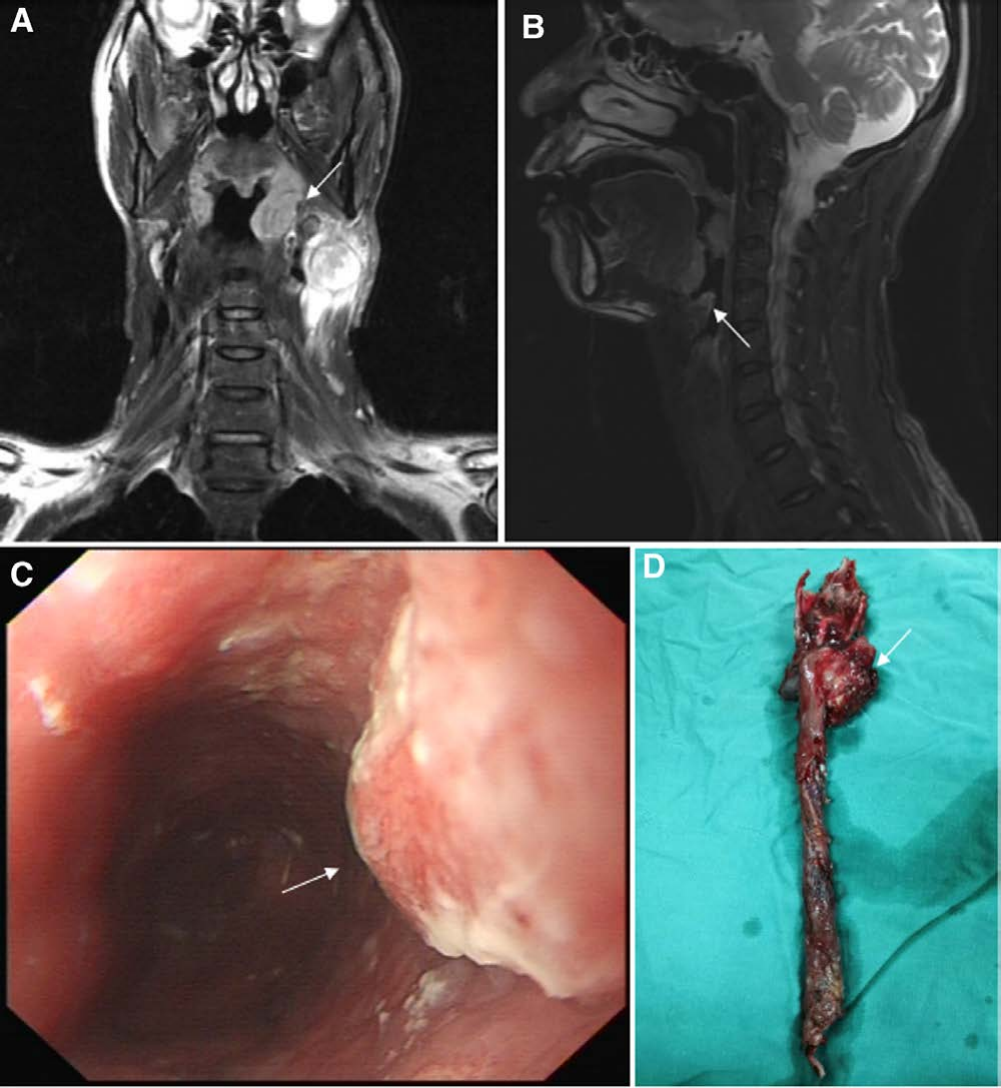
Imaging and operative findings in a patient with synchronous head-and-neck and esophageal cancer. (A and B) MRI images with arrows indicating cancer involvement in the left tonsil and epiglottis. (C) Gastroscopy image with an arrow highlighting the esophageal cancer. (D) Operative specimen comprising the left tonsil, epiglottis, hyoid bone, base of the tongue, upper thyroid cartilage, and esophagus.

A 51-year-old male, who had a history of smoking 20 cigarettes per day and drinking 10 g of alcohol per day for over 30 years, was admitted to the ENT department in October 2018 for a left neck mass with pharyngeal discomfort lasting more than 5 months. Following MRI, laryngoscopy, gastroscopy, and histopathological examinations, the patient was diagnosed with left tonsil cancer (Fig. 1A, squamous cell carcinoma, T2N2bM0), epiglottic cancer (Fig. 1B, squamous cell carcinoma, T1N2bM0), and lower esophageal cancer (Fig. 1C, squamous cell carcinoma, T2N0M0). He received chemotherapy with paclitaxel combined with cisplatin for the following month and underwent resection of the left tonsil, epiglottis, hyoid, tongue base, upper edge of the thyroid cartilage, and reconstruction of the esophagus with a gastric tube after esophagectomy (Fig. 1D). The patient was referred to the rehabilitation department with dysphagia on December 6, 2018.

## 3. Rehabilitation evaluation

The initial rehabilitation evaluation included swallowing screening, assessment of swallowing organs, videofluoroscopic swallowing study (VFSS), flexible endoscopic evaluation of swallowing (FEES), and nutritional assessment (Table 1).

**Table 1:** Evaluation of pre-intervention and post-intervention for the patient.

Outcome measure	Pre-intervention	Post-intervention
MBSImP	Score 39	Score 9
Functional Dysphagia Scale (FDS)	Score 79	Score 14
Dysphagia Outcome and Severity Scale (DOSS)	Score 1	Score 6
Rosenbek Penetration Aspiration Scale (PAS)	Score 8	Score 1
Functional oral intake scale (FOIS)	Score 1	Score 6
Fiberoptic Endoscopic Dysphagia Severity Scale (FEDSS)	Score 6	Score 1
Repetitive Saliva Swallowing Test (RSST)	0–1	2–3
Serum albumin (g/L)	30	37
Hemoglobin (g/L)	86	106
Skinfold Thickness of Triceps Brachii (mm)	8.0	13.2
BMI (kg/m^2^)	18.2	20.4

DOSS = Dysphagia Outcome and Severity Scale, FDS = Functional Dysphagia Scale, FEDSS = Fiberoptic Endoscopic Dysphagia Severity Scale, FOIS = Functional Oral Intake Scale, MBSImP = modified barium swallow impairment profile, RSST = Repetitive Saliva Swallowing Test.

### 3.1. Outcomes of the screening

The 10-item eating assessment tool score was 36. Using the volume-viscosity swallow test, swallowing function was assessed using the functional oral intake scale, in which the patient was placed at level 1, indicating no oral intake ability.

### 3.2. Assessment of swallowing organs

An evaluation of oral and facial functions showed that the tongue was atrophic, and the movement functions of the tongue and soft palate were weakened. Evaluation of the cervical muscle revealed that 2 surgical scars in the anterior region of the neck led to a decrease in muscle strength and joint range of motion. Evaluation of the swallowing reflex revealed that both the cough and pharyngeal reflexes were absent. Repetitive saliva swallowing test was performed to evaluate laryngeal function, and the results showed decreased larynx elevation and swallowing frequency.

### 3.3. VFSS

Using the modified barium swallow impairment profile to evaluate 17 physiological components of swallowing, we found that the patient’s total score was 39.

**Figure 2.**
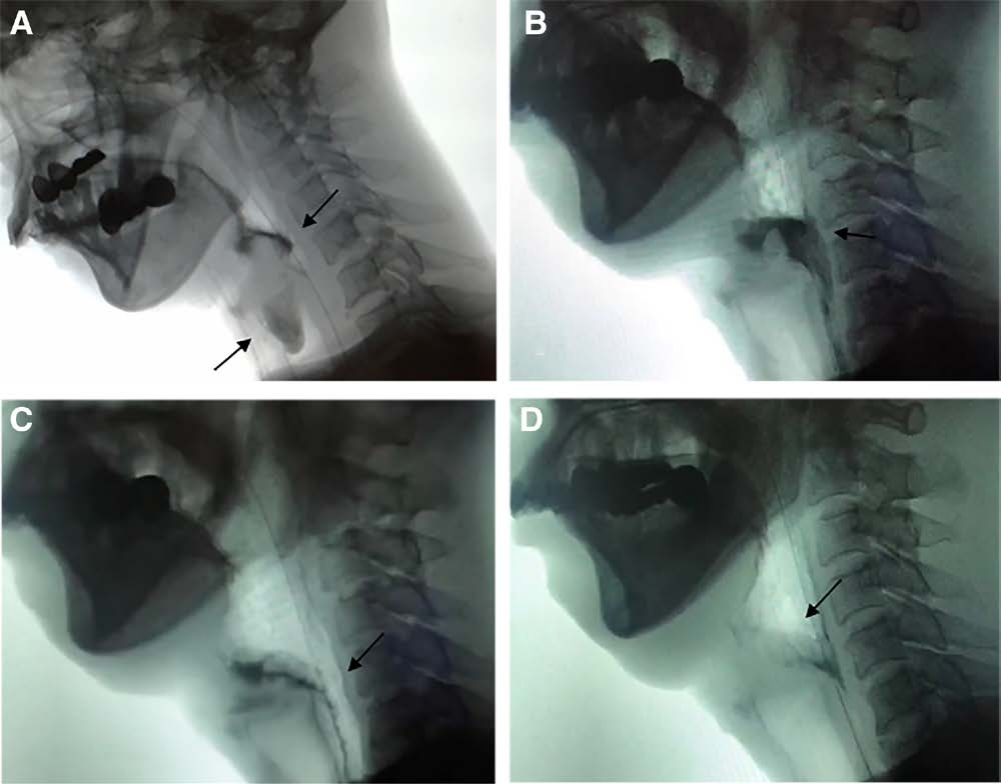
Videofluoroscopic swallow study (VFSS) of the patient. (A) Pre-therapy image showing aspiration of the bolus below the vocal cords (left arrow) and anastomotic stricture (right arrow). (B–D) Post-therapy images, with arrows indicating the anastomotic stricture site.

The Rosenbek Penetration Aspiration Scale produced a score of 8, indicating tracheal aspiration of material below the true vocal folds without effortful ejection (Fig. 2A). The Functional Dysphagia Scale was 79. The Dysphagia Outcome and Severity Scale showed a score of 1, indicating severe dysphagia.

VFSS further showed an anastomotic stricture at the level of 5th cervical vertebra (Fig. 2A). With a degree of narrowing severe enough to prevent even water from passing through.

### 3.4. FEES

**Figure 3.**
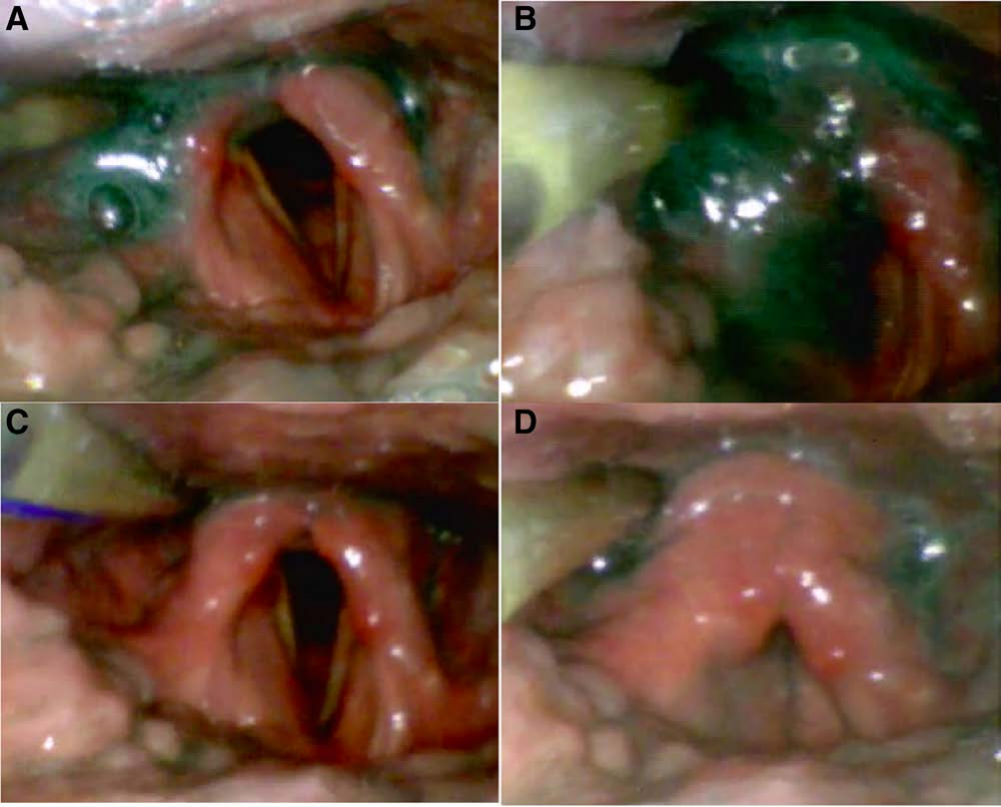
Fiberoptic endoscopic images of the patient. (A and B) Pre-therapy findings, and (C and D) post-therapy observations.

Using the FEES protocol, the score obtained on the fiberoptic endoscopic dysphagia severity scale was 6, and saliva pooling with aspiration was observed (Fig. 3A). The examination indicated the absence of epiglottis and hyoid, pharyngeal residue, material aspiration, and weakened movements of the aryepiglottic and vocal folds (Fig. 3B).

### 3.5. Nutritional assessment

An evaluation of the patient’s nutritional status showed that the serum albumin level was 30 g/L, the skinfold thickness of the triceps brachii was 8.0 mm, hemoglobin level was 86 g/L, and BMI was 18.2 kg/m^2^.

## 4. Rehabilitation therapy

The rehabilitation treatment was performed under the direction of a speech therapist for a duration of 5 days a week for 8 weeks.

### 4.1. Stretch training

The patient was instructed to stretch the muscle in the anterior neck daily to improve muscle strength and joint range of motion.

### 4.2. Swallowing behavior therapy

Swallowing therapy consisted of stimulation and exercises, including stimulation using ice and acid, oral and facial exercises, tongue base retraction exercises, tongue strengthening exercises, the Masako maneuver, the Shaker exercise, effortful swallowing, and the Mendelsohn maneuver.

The patient was instructed on how to perform the super-supraglottic swallow to resume oral feeding without aspiration.

### 4.3. Catheter balloon dilatation

The patient underwent repeated catheter balloon dilation for anastomotic stricture, 5 days a week for 2 months, and the amount of water injected into the balloon was gradually increased from 2 to 6 mL. In this process, the patient was asked to perform effortful swallowing and the Mendelsohn maneuver. Due to the severity of the stricture, which prevented the use of standard endoscopic balloon dilation, we opted for a smaller catheter balloon designed for children to treat the anastomotic stricture.

### 4.4. Electrical stimulation

The patient received VitalStim treatment for 20 minutes daily. The electrode placements were as follows: channel 1 just above the upper edge of the thyroid cartilage and channel 2 just below the upper edge of the thyroid cartilage.

### 4.5. Respiratory therapy

Respiratory therapy consisted of active cycles of breathing technique and conventional chest physical therapy, performed daily for the duration of the hospital stay.

## 5. Outcome

After 8 weeks of rehabilitation, the patient’s swallowing condition and nutritional status significantly improved (Table 1). He was able to take a puree orally without aspiration by performing a super-supraglottic swallow (Fig. 2B), and the esophagogastrostomy anastomosis was clear and free of obstruction (Fig. 2C), and the pharyngeal residue was negligible (Figs. 2D and 3C). Moreover, the movements of the aryepiglottic and vocal folds were almost fully recovered (Fig. 3D).

## 6. Discussion

The patient demonstrated dysphagia resulting from surgery for synchronous head-and-neck and esophageal cancer. The tumors were located in the oropharynx, larynx, and esophagus. The important physiological function of the head and neck structure determined that the goal of the treatment in such a patient was to improve the survival rate and preserve the function.^[3]^ The patient received a tracheotomy; resections of the left tonsil, epiglottis, hyoid, tongue base, and upper edge of the thyroid cartilage; and a reconstruction of the esophagus with a gastric tube after esophagectomy. Almost all aspects of the swallowing process remained dysfunctional postoperatively. Improving the swallowing function of patients in such a situation is conducive to enhancing their quality of life.

The tongue is an essential part of swallowing. Tongue pressure is generated by the contact between the tongue and palate, which pushes the bolus into the pharyngeal cavity. The key to pushing the bolus into the esophagus is the driving pressure produced by tongue base retraction.^[4]^ The oral and pharyngeal swallowing phase could be affected simultaneously after resection of the tongue base and tonsil, which is manifested in the prolongation and residue of the oral and pharyngeal phase. Among patients with oropharyngeal cancer, 80% suffer from dysphagia after cancer treatment, which has a significant impact on their quality of life. In this case, the patient underwent resection of the tongue base and hyoid bone, resulting in atrophy, limited tongue movement, and weak tongue base retraction. Tongue base retraction exercises and tongue strengthening exercises can improve tongue function, and effortful swallowing and the Mendelsohn maneuver are considered to increase tongue.^[5]^ The patient’s tongue function was largely recovered.

Bolus transit through the pharynx is likely facilitated by a combination of factors including tongue propulsion generated by tongue pressure and negative pressure within the upper esophageal sphincter (UES). Masako (1995) observed increased postoperative posterior pharyngeal wall movement in patients with anterior oral cancer and speculated that this was a compensatory movement. As a result, Masako developed a unique tongue-holding technique that could promote the movement of the posterior pharyngeal wall.^[6]^ In which case we used the Masako maneuver to strengthen the compensatory movement of the pharyngeal constrictor that was weakened after the operation.

The epiglottis is an important structural barrier that defends the airway and overturns to avoid aspiration of the bolus. In the present case, surgical removal of the epiglottis resulted in a high risk of aspiration. To resolve this problem, we instructed the patient on how to perform a super-supraglottic swallow, which is considered to be an effective defensive mechanism and is recommended for postoperative patients with laryngeal cancer. In addition, super-supraglottic swallowing is reported to enhance tongue pressure,^[7]^ which could further compensate for the defect in tongue function in this patient. With compensatory swallowing behavior therapy, such as effortful swallow and super-supraglottic swallow, the patient could finally swallow without aspiration.

The patient in this case had severe postoperative anastomotic stricture, which resulted in pharyngeal residue and aspiration. The incidence of anastomotic stricture after esophagogastrostomy is approximately 5% to 46%. It is conventionally used to treat anastomotic strictures using endoscopic balloon dilatation. However, as the diameters of different endoscopes range from 6 to 10 mm, they cannot pass through severe strictures with a diameter of <6 mm, thus limiting the use of endoscopic balloon dilatation in patients with severe strictures. In this case, we innovatively used a smaller catheter balloon dilatation designed for children to treat a postoperative anastomotic stricture. Catheter balloon dilatation is often used in the treatment of cricopharyngeal achalasia and anastomotic strictures. The potential mechanisms include stretching the smooth muscle fibers directly through the inflated balloon and stretching the sphincter to enhance sensory input and nerve plasticity.^[8]^ The diameter of a catheter used for children is only about 3 mm, which can pass through severe anastomotic strictures more easily. Moreover, it has the advantages of low cost, convenient operation, and less pain to the patient. In the course of balloon dilatation, the patient was asked to perform effortful swallowing and the Mendelsohn maneuver to improve their voluntary swallowing ability and their swallowing coordination.^[9]^ After performing catheter balloon dilation 40 times, the results of VFSS and FEES revealed that the anastomosis was unobstructed, allowing the bolus to pass without aspiration during a super-supraglottic swallow. The need for 40 dilation sessions was due to the severity of the postoperative anastomotic stricture. Unlike cricopharyngeal achalasia, which typically requires fewer treatments, patients with severe anastomotic stenosis may require significantly more interventions to achieve satisfactory results.

## 7. Conclusion

Dysphagia is a common complication of synchronous head-and-neck and esophageal cancers. Comprehensive rehabilitation evaluation and treatment of the swallowing process enable patients with synchronous head-and-neck and esophageal cancer to have safe oral intake after surgery.

## Author contributions

**Conceptualization:** Zhi-Yong Wang.

**Writing – original draft:** Xinyuan Xue, Amerull Azman, Cuicui Zhang, Yangjia Chen, Jun Ni, Zhi-Yong Wang.

**Writing – review & editing:** Xinyuan Xue, Amerull Azman, Cuicui Zhang, Yangjia Chen, Jun Ni, Zhi-Yong Wang.
